# HEC-Based Emulgels as Vehicles for NaDES Extracts: Safety, Efficacy, and Sensory Evaluation

**DOI:** 10.3390/gels12100870

**Published:** 2026-09-25

**Authors:** Milica Martinović, Vanja M. Tadić, Ana Žugić, Slavica Blagojević, Stevan Blagojević, Ivana Nešić

**Affiliations:** 1 Department of Pharmacy, Faculty of Medicine, University of Niš, Boulevard Dr. Zorana Djindjića 81, 18108 Niš, Serbia; ivana.nesic@medfak.ni.ac.rs; 2 Department for Pharmaceutical Research and Development, Institute for Medicinal Plant Research “Dr. Josif Pančić”, Tadeuša Koscuška 1, 11000 Belgrade, Serbia; azugic@mocbilja.rs; 3 Faculty of Pharmacy, University of Belgrade, Vojvode Stepe 450, 11221 Belgrade, Serbia; slavica.blagojevic@pharmacy.bg.ac.rs; 4 The Institute of General and Physical Chemistry, Studentski trg 12/V, 11158 Beograd, Serbia; sblagojevic@iofh.bg.ac.rs

**Keywords:** NaDES, Olivem^®^ 1000, sorbitan olivate, olivate-based emulsifier, alkyl glucoside, skin hydration, TEWL, emulgel, in vivo study, texture profile analysis

## Abstract

Natural deep eutectic solvent (NaDES) extracts exhibit substantial bioactive potential, including antioxidant, UV-protective, and enzyme-inhibitory activities, owing to their rich phytochemical composition. However, their incorporation into appropriate dermal delivery systems is essential to maximize their efficacy. Our previous studies identified emulgels as innovative and effective vehicles for NaDES extracts of green tea and bilberry leaves. In the present study, the in vivo performance of hydroxyethyl cellulose (HEC)-based emulgels containing 12% (*w*/*w*) NaDES extracts was evaluated. Formulations stabilized with cetearyl olivate/sorbitan olivate (5%) were compared with those stabilized using cetearyl alcohol/cetearyl glucoside (6%) in a study involving 25 healthy volunteers. Over a 28-day application period, key biophysical skin parameters, including skin pH, transepidermal water loss (TEWL), erythema index (EI), skin capacitance (EC), melanin index (MI), and friction coefficient (F), were monitored. Additionally, a 24 h skin safety assessment and a sensory evaluation were conducted. Both formulations demonstrated excellent safety profiles, with no signs of irritation observed during the 24 h safety study. The emulgels stabilized with cetearyl olivate/sorbitan olivate produced significant improvements in skin biophysical parameters. Skin barrier recovery, indicated by a reduction in TEWL, was achieved within two weeks, while enhanced skin hydration was maintained throughout the study period. Sensory evaluation further demonstrated superior spreadability of the olivate-based formulations. Overall, these findings confirm that HEC-based emulgels are promising dermal delivery systems for NaDES extracts, combining excellent safety, efficacy, and favorable sensory properties.

## 1. Introduction

Due to their stable three-dimensional hydrophilic polymer network, which enables retention of large quantities of water, hydrogels are at the center of attention when it comes to their application in the field of drug delivery systems, biomaterials, and cosmetics. Hydroxyethyl cellulose (HEC) is one of the most important gelling agents that can act as thickener, film-former, or stabilizer in pharmaceutical products. HEC is a well-studied, biocompatible, and low-cost gelling agent that does not cause immune reactions or toxicity, so it is often used as an alternative to synthetic polymers [1]. However, since stratum corneum is largely composed of lipids and hydrogels are vehicles limited in their ability to incorporate lipophilic substances, they are often reserved for preparations that are intended to form a film on the skin. By combining hydrogels with conventional emulsions, novel vehicles, emulgels, are formed, offering the advantages of both systems for topical administration [2,3]. Studies have shown that emulgels exhibit advantages regarding the stability of active substances, controlled release profile, administration of lower doses, low surfactant content, emollient but greaseless skin feel, favorable rheological and thixotropic behavior, and superior spreadability [3,4].

Alongside the use of innovative drug vehicles, the modern pharmaceutical industry is actively progressing toward a greener future. The incorporation of active ingredients of natural origin, the utilization of plant-based raw materials, cost-effectiveness, consideration of environmental impact, and minimization of waste generation represent current and future trends in the pharmaceutical industry. In that spirit, advancements in extraction technologies offer the opportunity to obtain “green extracts” characterized by superior quality, reduced energy consumption, minimal generation of toxic waste, and the elimination of hazardous solvents [5,6,7]. Natural deep eutectic solvents (NaDESs), as liquids composed of various biodegradable metabolites and natural compounds, are currently in the spotlight, with the intention to successfully replace dangerous solvents. The name itself indicates that the formed NaDES exhibits a lower melting point than its individual constituents. NaDESs were first noticed in plant metabolism, playing a biological role and bio-environment for metabolic processes [8,9,10].

In our previous studies, we formulated and characterized alpha-hydroxy-acid-based NaDES extracts of green tea leaves and bilberry leaves. By comparing their chemical compositions using spectrophotometric methods (determination of total phenolic and total flavonoid contents) and HPLC, we demonstrated that NaDES extracts exhibit a higher polyphenol extraction efficiency than conventional extracts (aqueous and ethanolic) [11]. This advantage in terms of chemical composition prompted us to evaluate their potential biological activities. Initially, we conducted in vitro assays, confirming their strong antioxidant and UV-protective effects, as well as their capacity to inhibit key skin enzymes, including hyaluronidase, collagenase, and tyrosinase [12]. Furthermore, we performed an in vivo study to assess the safety profile of these extracts incorporated into HEC-based gels, along with their anti-irritation potential against artificially induced skin irritation [13]. However, for any active substance to exert an optimal effect in a biological system, careful selection of a suitable vehicle is essential to ensure its stability, effective delivery, and bioavailability. The choice of vehicle is particularly critical for semi-solid dermal formulations, as the vehicle itself can influence skin properties and, consequently, the overall performance of the active substance. Therefore, in one of our previous studies, we investigated the selection of the most suitable vehicle for the incorporation and effective delivery of NaDES extracts. The results demonstrated that, compared to HEC-based gels, xanthan-gum-based gels, creams formulated with an olivate-based emulsifier, and xanthan-gum-based emulgels, the emulgel stabilized with an olivate-based emulsifier and HEC exhibited the most favorable performance [14]. Consequently, the new objective was set to evaluate these formulated emulgels in vivo on human volunteers to gain a comprehensive insight into their impact on skin biophysical parameters.

The primary aim of this study was to assess the safety of the emulgels using a 24 h occlusive patch test, which ensures continuous contact between the formulations and the skin and thus provides a sensitive assessment of their irritation potential. Additionally, the study evaluated the effects of 28 days of emulgel application on key skin biophysical parameters (electrical capacitance (EC) as an indicator of hydration, transepidermal water loss (TEWL) as an indicator of skin barrier integrity, erythema index (EI) as an indicator of skin redness and irritation, melanin index (MI) as an indicator of skin pigmentation, friction coefficient (F) as indicator of skin smoothness, and skin pH). The study also aimed to determine whether any effects persisted two days after discontinuation of treatment. In addition, sensory and textural analysis was conducted in order to characterize applicative characteristics of formulations. Furthermore, we aimed to compare the influence of the emulsifier choice stabilizing the emulgel system, specifically comparing Olivem^®^ 1000 (INCI: sorbitan olivate (and) cetearyl olivate), a newer and increasingly popular emulsifier emphasized in plant-derived formulations, with Montanov™ 68MB (INCI: cetearyl alcohol (and) cetearyl glucoside), a traditionally used and well-documented emulsifier.

## 2. Results and Discussion

### 2.1. In Vivo Safety Assessment

According to Regulation 1223/2009, a modern cosmetic product must be safe for application, making safety assessment a crucial aspect during its development. A cosmetic product must not pose any health risk to the user during daily application [15]. Therefore, as part of testing dermo-cosmetic preparations, so-called safety studies are conducted, among which the most common are studies evaluating the irritation potential of the applied preparation under occlusion.

The 24 h safety evaluation demonstrated that none of the tested emulgels caused adverse skin reactions. As part of these tests, parameters were measured at six sites on the forearms of volunteers, where one site was designated as an untreated control site (NC), one as an untreated control site under occlusion (NCO), while the tested emulgels were applied to the remaining four sites under occlusion using Parafilm and adhesive tape.

The results of measuring changes in TEWL (Figure 1), as an indicator of skin barrier integrity, showed that a statistically significant increase in this parameter occurred only at NCO (*p* < 0.05), at the untreated site subjected to occlusion for 24 h. In contrast, the OP, MP, and MA did not induce statistically significant changes in TEWL. The absence of statistically significant changes in TEWL at the treated sites indicated that irritation did not develop, as the applied emulgels mitigated the irritation caused by occlusion. At the site treated with the active emulgel OA, a statistically significant decrease in TEWL was observed (*p* < 0.05), indicating that this formulation not only failed to cause irritation but also effectively reduced transepidermal water loss through potential barrier support and/or vehicle occlusive effects.

These results were also confirmed by monitoring changes in EI (Figure 2), where a statistically significant increase in this parameter likewise occurred only at the NCO site (*p* < 0.05), whereas at the site treated with OA, a statistically significant decrease in this parameter was observed (*p* < 0.05).

Based on the changes in skin hydration level (Figure 3), a statistically significant increase in hydration (*p* < 0.01) occurred at all sites under occlusion, which was likely a consequence of hyperhydration caused by the occlusion itself. The increase in hydration was greater at all sites treated with one of the emulgels (OP, MP, OA, and MA) compared to NCO (*p* < 0.05), indicating a positive effect of the emulgel bases themselves on skin hydration. Nevertheless, direct post hoc comparisons showed no statistically significant differences between the sites treated with the four emulgels (Figure 3b), indicating that neither the emulsifier type nor the presence of the NaDES extract influenced the increase in EC.

Changes in pH were not statistically significant at any of the tested sites (Figure 4), confirming that surface skin pH remained within physiological boundaries.

Overall, the in vivo safety study demonstrated a favorable safety profile for all evaluated emulgels (OA, OP, MA, and MP). For the Montanov-based formulations (MA and MP), no significant differences in skin response were observed, indicating that the incorporation of NaDES extracts did not affect the short-term tolerability of these formulations. In contrast, unlike the other formulations, OA produced a significant reduction in TEWL and EI (*p* < 0.05), suggesting a potential additional soothing effect. However, this observation should be confirmed in further studies.

In a study involving 10 female volunteers with a mean age of 22.3 ± 0.6 years, the irritation potential of emulgels based on cetearyl alcohol and cetearyl glucoside (7%) was evaluated in a 24 h study under occlusion [16]. A decrease in TEWL was detected at the treated sites despite occlusion and a statistically significant increase in EC, while changes in EI were not statistically significant. The obtained results were in agreement with the findings of our investigation.

In a similar study evaluating the safety of a preparation also containing 7% of this emulsifier, no statistically significant changes in TEWL and EI occurred, whereas a statistically significant increase in EC was observed [17].

A literature search revealed no data on in vivo safety studies conducted to evaluate preparations stabilized by a self-emulsifying system composed of cetearyl olivate and sorbitan olivate.

### 2.2. In Vivo Long-Term Efficacy Assessment

The effects of long-term application of emulgels OP, OA, MP, and MA were investigated in a 30-day study. Changes in biophysical skin parameters were monitored after 7, 14, and 28 days of application compared to baseline values measured at the start of the study. Additionally, effects were evaluated two days after discontinuation of sample application. Healthy volunteers with no underlying skin conditions participated in the study.

Values of biophysical skin parameters can vary depending on skin health and environmental conditions, making it difficult to establish “normal” values for biophysical parameters, such as TEWL and EC. As a standard provided by the U.S. Food and Drug Administration (FDA), a TEWL < 15 [18] is used, which was consistent with the baseline values recorded across all skin sites of the participants.

Observation of changes in TEWL (Figure 5) revealed a statistically significant decrease in this parameter across all treated sites, as confirmed by a highly significant main effect of time (F(4, 64) = 8.392, *p* < 0.001, η_p_^2^ = 0.344). TEWL decreased throughout the study period, with the lowest values recorded after 28 days. The most substantial reduction in TEWL occurred after 7 days for the OA and OP emulgels, and after 14 days for the MA and MP emulgels, whereas across all formulations the differences between measurements on day 14 and day 28 were minimal and not statistically significant in any case. This result suggests that maximum TEWL reduction was achieved after two weeks, after which the values stabilize, implying that the skin adapts to the preparation. This outcome may indicate that the skin barrier was strengthening or that formulations have formed films and caused an occlusive effect on the skin. In addition, TEWL decreased with different dynamics, depending on the emulsifier used, which may be attributed to the liquid-crystalline structure present in samples MP and MA, delaying the onset of full efficacy. Furthermore, factorial analysis revealed a statistically significant interaction between emulsifier and extract (F(1, 16) = 12.044, *p* = 0.003, η_p_^2^ = 0.429), indicating that the influence of the NaDES extract on TEWL was strongly dependent on the vehicle used. For emulgel OA, a statistically significant difference in measured TEWL values was observed as early as day 7 (*p* < 0.01) and persisted at every subsequent measurement relative to baseline. The lowest mean TEWL values in the study were recorded precisely at the site treated with sample OA after 28 days, when the reduction in TEWL was most pronounced (ΔTEWL = −2.398, corresponding to a 32.76% decrease compared to initial baseline values at that site of 7.32 ± 2.33). At the final measurement, an increase in TEWL was observed across all treated sites as a result of treatment discontinuation. The return of TEWL toward baseline values after stopping application was more pronounced for the placebo samples, suggesting that the NaDES extracts may exert an additional effect in maintaining reduced water loss even after active treatment ceases, potentially through enhanced stratum corneum water-retention mechanisms alongside vehicle occlusion. Moreover, sample OA was the only formulation for which TEWL remained statistically significantly different from baseline two days after treatment cessation (day 30 of the study; *p* < 0.01). Given the significant emulsifier × extract interaction, this prolonged effect suggests that olivate-based vehicle facilitates the effect of active components from NaDES extracts.

Regarding the EC values (Figure 6), unlike the untreated control site, where changes were not statistically significant, a statistically significant increase in EC occurred at all other sites after just seven days (*p* < 0.01). Three-way RM-ANOVA confirmed an exceptionally strong main effect of time (F(4, 64) = 128.698, *p* < 0.001, η_p_^2^ = 0.889). EC values continued to rise across all treated sites up to day 14, after which they reached a plateau and remained virtually unchanged through day 28. This outcome directly aligns with the observed reduction in TEWL, as reduced TEWL actively supports water retention and stratum corneum hydration. Throughout the entire study period, EC values were statistically significantly higher than baseline values (*p* < 0.01). This effect persisted even two days after treatment discontinuation (day 30 of the study), indicating a prolonged hydrating effect of the emulgels. Furthermore, factorial analysis established a statistically significant main effect for extract (F(1, 16) = 4.903, *p* = 0.042, η_p_^2^ = 0.235), demonstrating that the application of active formulations resulted in overall higher EC values than their respective placebo counterparts, confirming an additive hydrating effect of the NaDES extracts. Numerically, the most pronounced hydrating effect was measured at the sites treated with emulgel OA, which achieved the highest mean increase in hydration relative to baseline after 14 and 28 days (ΔEC = 23.55, representing a 71.35% increase compared to the initial baseline values at that site of 33.01 ± 6.59), as well as the greatest residual effect after 30 days (ΔEC = 9.05). The statistical analysis also revealed a significant three-way interaction, emulsifier × extract × time (F(4, 64) = 3.319, *p* = 0.016, η_p_^2^ = 0.172), which indicates that the influence of NaDES extract on hydration was different in each vehicle type, with olivate-based proving to be more effective.

Changes in F often correlate with variations in skin hydration [19], as was also observed in the present study. A statistically significant increase in F occurred at all treated sites as early as seven days after the start of the study, which was confirmed by statistical analysis indicating the significance of the effect of time (F(4, 64) = 34.267, *p* < 0.001, η_p_^2^ = 0.682). Maximum values were recorded after 14 days (Figure 7), after which they decreased. Neither the type of emulsifier used to stabilize the tested emulgels (*p* = 0.446) nor the applied extracts (*p* = 0.358) had a statistically significant effect on changes in this parameter. However, factorial analysis revealed a significant two-way interaction of emulsifier x extracts (F(1, 16) = 7.208, *p* = 0.016, η_p_^2^ = 0.311), suggesting that the impact of NaDES extracts on skin friction was dependent on the vehicle. The highest mean value of F and highest numerical overall increase (ΔF = 277.12) was measured after 14 days at the site treated with emulgel OA, where the combined effect of NaDES extract and vehicle was most pronounced.

In the case of EI (Figure 8), as expected, there were no statistically significant changes at the control site. A decrease in this parameter’s values was observed across all treated sites over time (F(4, 64) = 17.710, *p* < 0.001, η_p_^2^ = 0.525). For emulgels OA and OP, a statistically significant reduction in EI occurred as early as day 7 (*p* < 0.01), whereas for emulgels MA and MP, this effect was observed only after 14 days (*p* < 0.01). Similar to EC and TEWL, a plateau for this parameter was also noted after 14 days. The highest numerical reduction in EI compared to baseline values was measured after 14 days at the site treated with emulgel OA (ΔEI = 36.21). Factorial analysis confirmed higher ΔEI values for the active samples containing NaDES extracts compared to the placebo samples through a statistically significant two-way interaction, extract x time (F(4, 64) = 3.293, *p* = 0.016, η_p_^2^ = 0.171). In addition, a significant three-way interaction of emulsifier × extract × time (F(4, 64) = 3.708, *p* = 0.009, η_p_^2^ = 0.188) showed that the presence of NaDES extracts and the choice of emulsifier had a combined effect on EI.

A statistically significant decrease in MI was observed at the sites treated with the active emulgels (*p* < 0.05), but not at those treated with the placebo preparations (*p* > 0.05; Figure 9), indicating that the presence of the NaDES extracts as active cosmetic ingredients contributed to this effect, emulsifier × extract (F(4, 64) = 49.737, *p* < 0.001, η_p_^2^ = 0.757). In a previous study, the selected extracts demonstrated the potential to exhibit antityrosinase activity [12], which can explain the obtained result. Post hoc comparisons confirmed that no significant difference in the MI reduction was observed between the two active formulations OA and MA (*p* > 0.05).

As one of the essential indicators of skin condition, pH is a parameter whose change following the application of a tested formulation may point to a disruption of the skin’s hydrolipid film [20]. Therefore, applied preparations must not disturb the “normal” physiological skin pH. Although minor statistically significant changes in skin surface pH were observed across certain time points (Figure 10), all measured values remained strictly within the “normal” physiological range (pH 4.0–6.0) throughout the study.

Previous studies have demonstrated the beneficial effects on the skin of preparations containing extracts of the utilized herbal drugs. A cosmetic product containing bilberry seed oil (6%) obtained by supercritical extraction and ethanol bilberry leaf extract (6%) obtained by maceration showed the potential to increase stratum corneum hydration and decrease TEWL in a long-term 28-day study [21]. Incorporating green tea leaf extract into a formulation at a 6% concentration was also shown to influence skin hydration, exerting a prolonged moisturizing effect and improving skin microrelief after 15 days of application [22]. Following five weeks of application of a preparation containing green tea leaf extract, a more pronounced increase in hydration (*p* < 0.05) was observed at treated sites compared to sites treated with a vitamin E formulation [23]. Emulgels containing 20% commercial green tea leaf extract induced a statistically significant increase in hydration and a reduction in TEWL after five days of sample application [24]. Improvement of skin barrier function was further confirmed in an 8-week study in which 10 male volunteers applied formulations containing ethanol green tea leaf extract (3%) obtained by maceration [25].

NaDES extracts used in this study were characterized in our previous studies where their chemical composition was confirmed, followed by the evaluation of their in vitro and in vivo biological activities [11,12,13,14]. It is important to note that the observed enhancement in skin biophysical parameters for the active emulgels OA and MA reflects the combined effect of the integrated NaDES–phytochemical complex (comprising both the extracted secondary metabolites and the constituents of the NaDES vehicle, e.g., polyols and organic acids). Given that components such as glycerol and sorbitol are established humectants that interact directly with stratum corneum hydration and barrier restoration [26], the NaDES vehicle acts not merely as an inert solvent, but as a functional co-active agent alongside the plant secondary metabolites. Nevertheless, the specific biological contribution of the extracted plant constituents is strongly supported by our previous in vitro investigations comparing pure (blank) NaDES systems with their corresponding NaDES plant extracts. In those studies, the presence of the extracted phytochemical matrix yielded significantly superior bioactivity compared to the solvent alone, particularly in terms of UV-protective capacity and the inhibition of key dermal enzymes, including hyaluronidase, collagenase, and tyrosinase [12]. Therefore, while the NaDES vehicle inherently provides hydration and barrier support, the integrated NaDES–phytochemical complex operates as a unified, synergistic system that maximizes both biophysical and biological skin benefits.

The emulsifier consisting of a mixture of cetearyl olivate and sorbitan olivate was used at an 8% concentration in creams formulated with urea and sodium hyaluronate. In patients with xerosis, a condition characterized by severely dry skin, this formulation significantly reduced TEWL after 28 days of application [27].

In the study, for the preparation of OA and OP, a dual olivate system compliant with COSMOS standards and aligned with green chemistry principles was employed. The system consisted of Olivem^®^ 1000 (INCI: cetearyl olivate, sorbitan olivate) and Olivem^®^ VS Feel (INCI: cetearyl alcohol, cetyl palmitate, sorbitan palmitate, sorbitan oleate). Both cetearyl olivate and sorbitan olivate are derived from olive oil fatty acids and exhibit structural affinity for skin surface lipids, due to their similarity to the stratum corneum lipid composition and their ability to form liquid-crystalline structures. Based on literature-supported data for these emulsifying systems, it is hypothesized that the formation of lamellar assemblies resembling endogenous intercellular lipid bilayers may facilitate lipid matrix reorganization and repair, which might partly explain the observed reduction in TEWL [28,29,30].

Montanov™ 68MB (INCI: cetearyl alcohol (and) cetearyl glucoside), an alkyl-glucoside-based emulsifier, also forms lamellar liquid crystals; however, its crystalline organization differs because of their distinct chemical composition. While cetearyl alcohol is responsible for a denser lattice structure, sorbitan olivate induces a higher degree of molecular parallel packing within the liquid-crystal phase [31].

The liquid-crystalline architecture reported for these types of vehicles provides a possible explanation for why skin hydration remained significantly higher than baseline values (*p* < 0.05) for all emulgels, even two days after treatment cessation. As suggested in the literature, the lamellar network is thought to act as a sustained water reservoir, providing prolonged hydration through controlled water retention. Nevertheless, one of the primary reasons for the observed differences between alkyl-glucoside-based emulgels and olivate-based emulgels is suggested to lie in the chemical structure of their constituents. While cetearyl olivate and sorbitan olivate are derived from an unsaturated fatty acid (oleic acid), cetearyl alcohol and cetearyl glucoside are based on saturated C16–C18 fatty alcohols. The saturated alkyl chains tend to form more rigid interfacial structures that remain primarily on the skin surface, while oleic-acid-derived components may interact more readily with stratum corneum lipids because of their greater molecular flexibility and penetration potential [32,33]. Although these compositional differences provide a plausible explanation for the distinct TEWL reduction profiles observed between the two emulsifying systems, further structural characterization of their liquid-crystalline organization is needed to elucidate their stabilization mechanisms.

The contribution of the co-emulsifying system should also be considered when interpreting the superior performance of the olivate-based formulations. Olivem^®^ 1000 is reported to function as the primary lamellar liquid-crystal emulsifier, establishing the interlamellar network responsible for water retention, whereas Olivem^®^ VS Feel is described as an olive-derived biomimetic co-emulsifier that can enhance lipid fluidity, improve interactions with the skin hydrolipidic film, and contribute to increased skin hydration and elasticity. In addition, Olivem^®^ VS Feel improves the stability and viscosity of oil-in-water systems while serving as a sensorial modifier, thereby contributing to the overall performance of the formulation [34].

### 2.3. Sensory Analysis

Sensory analysis describes the relationship between a product and its perception and evaluation through the senses. Sensory characteristics represent key quality criteria for cosmetic formulations, directly influencing user perception and product acceptability. Their importance is further highlighted by recent neuroscience studies on emotionally driven decision-making processes in humans, which have shown that initial application feel is particularly critical for topical formulations, as satisfactory sensory attributes ensure high user adherence to application regimens. Therefore, sensory testing methods involving trained panelists and untrained consumers represent essential tools that address key questions across product development, quality control, marketing, and sales sectors [35].

The results of the sensory study conducted as part of this study showed that there were certain differences in how the emulgels were perceived by the panelists, though in most cases these differences were minor. Panelists received instructions to evaluate the properties of the emulgels via a questionnaire prior to application, during the application process, and 10 min post-application.

When observed prior to application, all emulgels were evaluated as semi-solid and shiny (Figure 11).

During the application, the panelists evaluated the emulgels differently depending on the used emulsifier (Figure 12). OP and OA were rated as preparations that exhibit very good spreadability. Compared to MP and MA, the OA and OP emulgels were perceived by the panelists as easier to spread, faster to absorb, and leaving a less greasy residue as well as less skin shine. Emulgel OP was evaluated as the least sticky preparation, whereas the remaining emulgels were rated as slightly sticky. All emulgels were predominantly described as slightly thick and slightly greasy. Emulgel MP was rated as the preparation leaving the most greasy and shiny residue on the skin, as well as having the slowest perceived absorption rate. On the other hand, emulgel OA was evaluated as the fastest-absorbing preparation, leaving the least shiny and greasy residue. Emulgel MA was rated as the thinnest preparation, while MP was rated as the thickest. It should be noted that the parameter evaluated by the participants as ‘absorption rate’ strictly reflects their subjective perception of the formulation’s disappearance from the skin surface (i.e., perceived drying time or residue reduction) rather than actual cutaneous or transdermal absorption, which cannot be quantified through sensory evaluation.

Ten minutes post-application (Figure 13), emulgels MP and MA were evaluated as leaving a greater amount of residual film than OA and OP. While over 50% of the participants assessed that emulgels OA and OP left no film on the skin, in the case of MP and MA, over 50% of the participants evaluated that a moderate film remained on the skin. Accordingly, the panelists rated the skin as greasier following the application of emulgels MP and MA than after the application of emulgels OP and OA. According to the panelists’ evaluation, the skin was least sticky following the application of emulgel OP and most sticky after the application of emulgel MP, which is consistent with the panelists’ observations during sample application itself.

According to the majority of the panelists, emulgel OA was selected as the most preferred formulation by a majority vote (Figure 14).

### 2.4. Texture Analysis

Texture analysis, as an objective method for analyzing the mechanical properties of topical preparations, was conducted to compare the textural characteristics of emulgels based on both emulsifiers used in the study. The results (Table 1) showed that there were differences and similarities in certain textural characteristics among the tested emulgels.

The results showed that the addition of NaDES extracts led to an increase in emulgel hardness for both emulsifiers used (*p* < 0.05), which can be partially explained by the higher adhesiveness of samples MA and OA. Sample OA exhibited the highest adhesiveness, which may indicate potentially better skin adhesion, whereas sample OP demonstrated the lowest adhesiveness (*p* < 0.05). These results align with the results of the sensory study, where panelists rated sample OP as the least sticky compared to the other preparations.

Additionally, higher hardness indicates poorer spreadability. It was observed that M samples had higher hardness values compared to O samples in the case of both placebo and active emulgels (*p* < 0.05), which may suggest better spreadability of O samples. These observations are consistent with the sensory study findings, where panelists marked the O emulgels as easier to spread compared to the M formulations.

The analysis of results one month after storage indicated an increase in hardness across all emulgels (*p* < 0.05). A similar trend was observed for cohesiveness (*p* < 0.05), pointing to a strengthening of the emulgel structure over time. A potential underlying reason for this phenomenon could be the additional structuring of the emulgels resulting from the expansion of the gel network post-gelling [36].

In addition to hardness, adhesiveness, and cohesiveness, springiness and resilience were also evaluated as part of the texture analysis, as similar parameters that nevertheless differ in key aspects. Resilience indicates the ability of the product to recover its original state after deformation and is calculated as the ratio of energy during the decompression phase to energy during the compression phase of the first compression cycle. On the other hand, springiness is evaluated after the second compression cycle and represents the ratio of the product height after the second cycle relative to the initial height. While resilience relates to the energy required for elastic recovery, springiness describes the difference in product height before and after deformation [37]. The results showed that no changes in resilience occurred over time for any of the tested emulgels (*p* > 0.05). Springiness decreased slightly in almost all samples, with the only statistically significant decrease measured in MP (*p* < 0.05).

Overall, the combined findings from the instrumental texture profile analysis and the sensory evaluation show a consistency between objective mechanical parameters and subjective consumer perception of emulgels. The lower hardness and adhesiveness of the olivate-based emulgels (OA and OP) aligned with superior spreadability, faster perceived skin absorption, and a lighter, non-sticky application feel compared to MA and MP. Consequently, formulation OA was rated as the overall favorite emulgel by the panelists. The focus of our future work would be to conduct a detailed rheological profiling of these emulgels in order to further study their flow behavior and structural viscoelasticity.

## 3. Conclusions

Self-emulsifying systems stabilized with 5% cetearyl olivate and sorbitan olivate (OA and OP) or 6% cetearyl alcohol and cetearyl glucoside (MA and MP) demonstrated a good safety profile, with no adverse skin reactions observed in the in vivo irritation potential study. Four-week application of the tested formulations resulted in a significant improvement in key skin biophysical parameters (EC, TEWL, EI, and F). All formulations exhibited a rapid and sustained hydrating effect that was statistically significant after 7 days of application and persisted throughout the entire 30-day study period, including the post-application phase. The greater increase in skin hydration and the reduction in melanin index observed for the active formulations (OA and MA) compared with their respective placebo formulations suggested an additional beneficial contribution of the NaDES plant extracts to these biophysical effects. Changes in the friction coefficient were consistent with changes in skin hydration.

Among the tested formulations, OA exhibited particularly favorable overall efficacy. It demonstrated a rapid improvement reduction in TEWL, as well as a decrease in erythema index, both of which were statistically significant after only seven days of application. These effects persisted for two days following treatment cessation.

Sensory analysis results demonstrated that the application properties and sensory profiles were influenced by both the emulsifier and the incorporated extract. Formulations stabilized with cetearyl/sorbitan olivate (OA and OP) exhibited superior spreadability, a faster perceived absorption rate, and less residual greasy film compared to systems formulated with cetearyl alcohol/glucoside (MA and MP). Among all tested formulations, OA was the most preferred by the majority of panelists. Furthermore, texture profile analysis aligned with the results of sensory evaluation findings and confirmed the structural integrity of the emulgels over 30 days of storage.

## 4. Materials and Methods

### 4.1. Materials

The plant materials used for the study are portrayed in Table 2.

Extraction solvents (NaDES) were made using tartaric acid and malic acid (Centrohem, Stara Pazova, Serbia) and sorbitol and glycerol (Comcen, Belgrade, Serbia).

Emulgels were prepared using the raw materials shown in Table 2.

### 4.2. NaDES Preparation

The NaDESs used in the study were malic acid-glycerol (1:2 mol/mol; MG) and tartaric acid-sorbitol (1:2 mol/mol; TS). Separate components were mixed for 30 min while heating on a magnetic stirrer (IKAMAG, IKA, Verke, Staufen, Germany), until clear NaDESs were formed. For correction of viscosity, distilled water was added at 30% (*v*/*v*). The structure of NaDESs was previously confirmed using Fourier transform infrared spectroscopy (FTIR) on an Agilent Cary 630 FTIR Spectrometer (Agilent Technologies, Inc, Santa Clara, CA, USA) [13].

### 4.3. NaDES Extracts Preparation

The ultrasound-assisted extraction at 50 °C in a sonication water bath (Gesellschaft fur Labortechnik, Burgwedel, Germany), as one of the most common methods of NaDES preparation, lasted for 30 min. The water bath operated at a standard ultrasonic frequency of 40 kHz and an effective power of 120 W. The mass ratio of dried powdered plant material (BL and TL) and NaDESs (MG and TS) was 1:20. After the extraction, the extracts BL-MG and TL-TS were centrifuged (6000 rpm for 10 min, laboratory Centrifuge LC 320, Tehtnica, Železniki, Slovenia) for separating the plant residues, and the supernatant was used as an active ingredient for emulgel preparation. The chosen extracts BL-MG and TL-TS were standardized based on their active compound profile, as characterized in our previous studies (chlorogenic acid and epigallocatechin) [11,13], ensuring consistency.

### 4.4. Emulgel Preparation

Four emulgels were prepared (Table 3):

- Placebo emulgel with cetearyl alcohol and cetearyl glucoside (MP).
- Active emulgel with cetearyl alcohol and cetearyl glucoside (MA).
- Placebo emulgel with sorbitan olivate and cetearyl olivate (OP).
- Active emulgel with sorbitan olivate and cetearyl olivate (OA).

The primary step in emulgel preparation was the formulation of the hydrogel base. The water phase consisting of water, glycerol, and preservative was gelled at room temperature by dispersing the gelling agent (HEC). HEC was allowed to hydrate for 1 h while mixing on a RW 16 basic propeller rotary laboratory stirrer (IKA Werke, Staufen, Germany). The oil phase, containing caprylic/capric triglycerides and emulsifier, was separately prepared. Both phases were independently heated to 70 °C on two laboratory magnetic stirrers (IKA C-MAG HS 7, Staufen, Germany). The heated water phase was then slowly added to the melted oil phase under continuous stirring at 500 rpm for 15 min using a propeller laboratory stirrer [38]. After cooling to a temperature below 40 °C, NaDES extracts were incorporated in active emulgels (MA and OA) and homogenized for 10 min. Unlike active emulgels (MA and OA), placebo emulgels (MP and OP) contained water instead of extracts. Water lost due to evaporation was compensated with distilled water to adjust to the final batch weight. The concentrations of the NaDES extracts and emulsifiers were selected based on preliminary pilot studies and preformulation screening. The NaDES extract concentration was chosen in accordance with the results of our previous study, in which the emulgels with 12% of NaDES demonstrated optimal biological activity and dermal suitability [14]. The emulsifier concentrations were selected within the manufacturer’s recommended limits after preformulation studies [29,39].

### 4.5. In Vivo Study

Healthy volunteers with no prior skin diseases were enrolled in the study. All participants were instructed to refrain from using topical products on the forearms for 14 days before the study and through the entire study period. All participants signed written informed consent after being fully informed about the aims and the protocol of the study. The study was conducted in accordance with the Declaration of Helsinki and was approved by the Ethics Committee of the Faculty of Medicine, University of Niš (Approval No. 12-10650/2-6, 3 October 2022).

To eliminate carryover effects and potential subject bias between study phases, 3 independent cohorts of healthy volunteers were separately recruited for the 24 h safety evaluation, the 28-day efficacy study, and the sensory analysis, respectively.

The number of participants was estimated using the G*Power 3.1 software by applying the parameters in the program (Table 4) after choosing option ANOVA: repeated measures, within-factors design. The effect size was defined according to Cohen’s conventions for an effect size, and type I (α) and type II (β) error probability were set as standard thresholds for statistical hypothesis testing. Since all participants applied all formulations and controls on their forearms, and thus served as their own control, the number of groups was set to 1. The number of measurements was defined based on the number of time points in the study protocols (2 for the safety study and 5 for the long-term study). Sample size calculations indicated that a minimum of 15 volunteers was required for the short-term safety assessment, whereas at least 11 volunteers were necessary to evaluate long-term efficacy.

The application site for each formulation was randomly assigned. Both the participants and the investigator conducting the study remained blinded to the identity of the applied emulgel until the study was completed. After the data analysis, the allocation of application sites (LU—left upper, LM—left middle, RU—right upper, and RM—right middle) to the corresponding formulations (MA, OA, MP, and OP) was unblinded (Figure 15). The precise application sites were defined using a custom-designed cardboard template.

For the non-invasive in vivo measurements, the device Multi Probe Adapter System MPA^®^ 9 (Courage + Khazaka Electronic GmbH, Germany) was used. The probes attached to the device are stated in Table 5.

The room temperature, monitored by the RHT 100 Ambient Condition Sensor, was set to 22 ± 1 °C, while humidity was 45 ± 5%. Before each session, volunteers rested with exposed forearms for 30 min to facilitate acclimatization and minimize environmental effects on skin parameters. This protocol adheres to guidelines set by the European Group for Efficacy Measurement of Cosmetics and Other Topical Products (EEMCO) [40].

#### 4.5.1. In Vivo Safety Assessment

To evaluate the safety profile of the emulgels, a double-blind study with 20 healthy volunteers of both genders (25.13 ± 4.87 years) was performed. Emulgel samples were applied at a dosage of 0.016 g/cm^2^ to designated sites on the volar forearm of each subject. The next step involved covering of treated skin areas with an occlusive film (Parafilm^®^, American National Can. Co., Chicago, IL, USA) and fixing the occlusion for 24 h with self-adhesive tape (Omnifix^®^ E, Hartmann, Heidenheim, Germany). Besides emulgel-treated sites, two control sites were included. NC represented a non-treated control site where no occlusion was applied, while NCO, non-treated control under occlusion, was a site covered with the occlusive film and adhesive tape where no sample was applied.

Skin parameters (EC, TEWL, EI, and pH) were recorded before the study began (for obtaining baseline values) and 60 min after the removal of 24 h occlusion.

#### 4.5.2. In Vivo Long-Term Efficacy Assessment

A double-blind study on 25 healthy volunteers of both genders (30.9 ± 10.21 years) was applied for long-term efficacy assessment of emulgels. Unlike the safety study, where the emulgels were applied only once by the investigators, participants in the long-term study were asked to apply the formulations twice daily (in the morning and in the evening) to predefined skin sites (Figure 15) for 28 consecutive days. To ensure consistent application-site identification, each participant received a cardboard template with a 3 × 3 cm cutout. The first application was performed under investigator supervision to demonstrate the correct application technique using a standardized fingertip unit dosage. Participant compliance was monitored throughout the study using daily application checklists and was verified at the end of the study by inspecting all returned formulation containers.

Five measurements of EC, TEWL, EI, MI, F, and pH were conducted during the study–basal measurement (before application of samples), measurements on day 7, on day 14, and on day 28. The last measurement of biophisical skin parameters was conducted 2 days after the end of the 28-day application period, i.e., on the day 30 when the aim was to measure the prolonged effect that persists after stopping the emulgel application. In addition, one site was left as a non-treated control (NC) for monitoring parameters of untreated skin.

### 4.6. Sensory Analysis

Sensory analysis was conducted in a room with good lighting and normal ambient conditions (air temperature 21 ± 2 °C, relative humidity 45 ± 3%). At the beginning of the study, the participants were fully introduced to the concept and protocol of the research, as well as the method for evaluating the sensory attributes. The participants were given full instructions on how to apply the preparations during the course of the study and at what quantity (standardized fingertip unit dosage). Like in the long-term in vivo study, they were given the packages with the notes of the location of the application on the forearm. To ensure objectivity, a single-blind, coded study design was implemented. The encoding of the emulgel containers was performed by an independent individual not involved in the execution of the study or data analysis; thus, neither the participants nor the investigators were aware of the specific formulation contents. While participants were informed of the general ingredient list, they were blinded to the specific contents of each container. Although subtle differences in physical appearance and color were visible among the formulations, participants had no prior knowledge regarding which visual characteristics corresponded to the placebo or active emulgels. The participants (32 participants, mean age 29.15 ± 11.18 years) completed a questionnaire assessing the sensory characteristics of the emulgels (Table 6) before, during, and after application. The questionnaire had been used in previous studies [21] and contains questions about sensory characteristics, with the list of descriptive terms.

The results obtained from the sensory questionnaire were analyzed descriptively. In order to avoid overly complex and difficult to follow interpretation of results, responses were first assigned quantitative numerical values and the results were converted into a quantitative scale (Table 7).

Then, a weighted average score was calculated for each attribute based on response proportions and linearly converted to a standardized 0–10 scale according to the following equation:

$$S_{10}=\left(\sum_{i=1}^{k}P_{i}\times i\right)\times \frac{10}{k}.$$

*S*_10_ is the final converted score on the 0–10 scale, *k* is the maximum number of response options for a given attribute (*k* ∈ {2, 3, 5}), *i* is the numerical rank assigned to each response option *i* = 1, 2 … k, and *P_i_* is the proportion of participants who selected response option *i* ($\sum P_{i}=1$).

In this way, overly complex reporting of categorical percentages across attributes evaluated on different ordinal scales (2 to 5 response options) is avoided and we were able to directly compare the results of sensory analysis visually on unified radar charts. No inferential parametric tests (e.g., ANOVA) were applied to these transformed descriptive values, and comparisons among formulations reflect descriptive trends in panelist evaluation.

### 4.7. Textural Analysis

Texture profile analysis (TPA) was performed using a CT3 Texture Analyzer (Brookfield, AMETEK Inc., Middleborough, MA, USA). The technique utilizes a double-penetration cycle with a conical probe (TA-STF) to mimic the action of a human finger scooping a formulation from its container. Samples were carefully transferred into the testing vessel to fill 75% of its capacity while avoiding air entrapment. Upon reaching a trigger load of 20 mN, the probe penetrated the sample to a target depth of 5 mm at a test speed of 1 mm/s. Key textural parameters, hardness, cohesiveness, adhesiveness, springiness, and resilience were recorded. All assays were conducted in triplicate at ambient temperature twice—after the emulgel preparation and after 30 days of storage.

### 4.8. Statistical Analysis

Results from the in vivo study were presented as mean ± standard deviation, as well as absolute changes (Δ) in measured parameters relative to baseline values at each assessment time point. Statistical comparisons in the long-term in vivo study were performed using three-way repeated-measures ANOVA following a 2 × 2 × 5 factorial design of factors: emulsifier—sorbitan/cetearyl olivate or cetearyl alcohol/glucoside × extracts, indicating presence of extract—placebo or active emulgel × time—basal, day 7, day 14, day 28, and day 30. Mauchly’s test of sphericity was applied to evaluate the assumption of sphericity. Whenever sphericity was violated (*p* < 0.05), degrees of freedom were adjusted using the Greenhouse–Geisser correction. Post hoc multiple comparisons between formulation groups and across time intervals were performed using Bonferroni’s test. The complete statistical parameters of three-way repeated-measures ANOVA were deposited in the Supplementary Material (Table S1). For the in vivo safety study, with comparisons of measured values for the same sample, for which only two measurements were conducted, Student’s paired *t*-test was performed. To compare changes between different treated sites and control sites in the safety study, one-way ANOVA with the appropriate post hoc test (Tukey’s test) was applied. One-way ANOVA with Tukey’s post hoc test was also used for independent assessment of texture analysis results between different emulgels, while Student’s paired t-test was used for evaluating texture parameter changes after 30 days. Statistical significance for all tests was established at *p* < 0.05. Statistical analysis was carried out using IBM SPSS Statistics 20, while diagrams were created using Microsoft Excel 10.

## Figures and Tables

**Figure 1:**
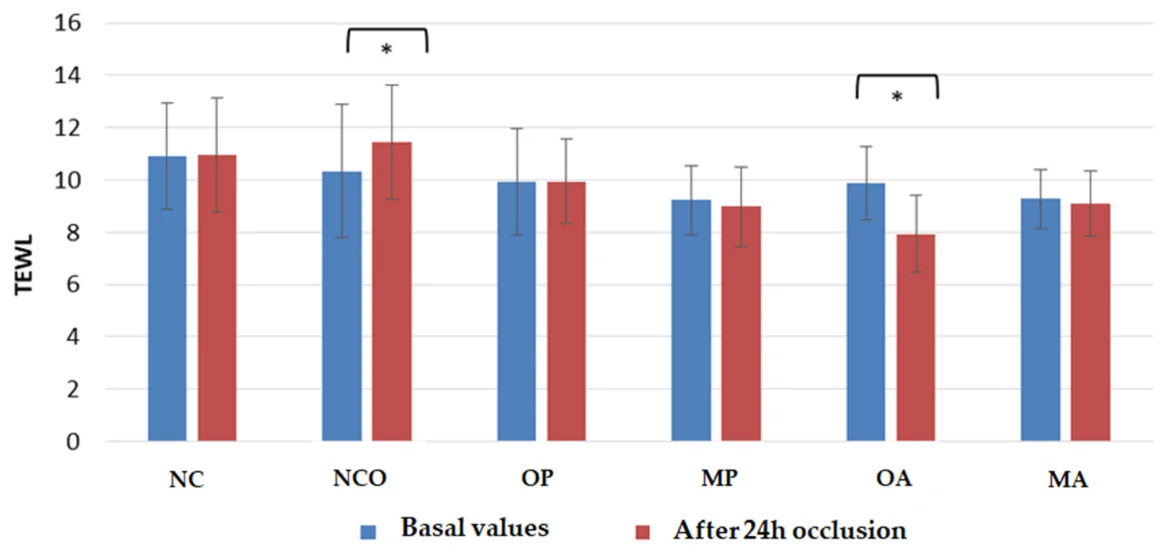
Absolute values of TEWL recorded during an in vivo study evaluating the irritation potential of emulgels (OP, MP, OA, and MA) and untreated controls (NC and NCO). Results are presented as mean ± standard deviation, with statistically significant differences marked by * (*p* < 0.05).

**Figure 2:**
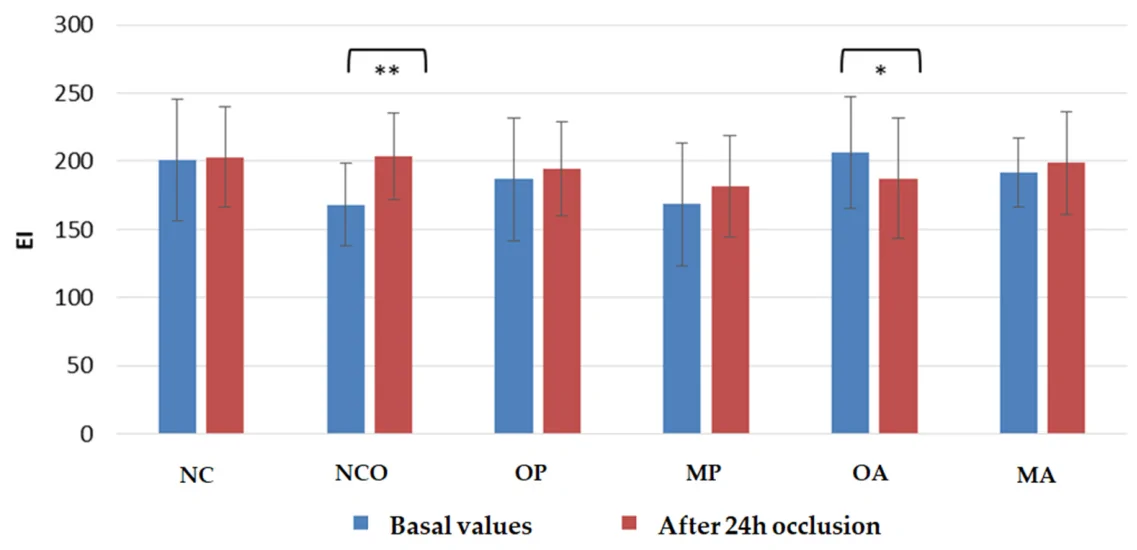
Absolute values of EI recorded during an in vivo study evaluating the irritation potential of emulgels (OP, MP, OA, and MA) and untreated controls (NC and NCO). Results are presented as mean ± standard deviation, with statistically significant differences marked by symbols * (*p* < 0.05) and ** (*p* < 0.01).

**Figure 3:**
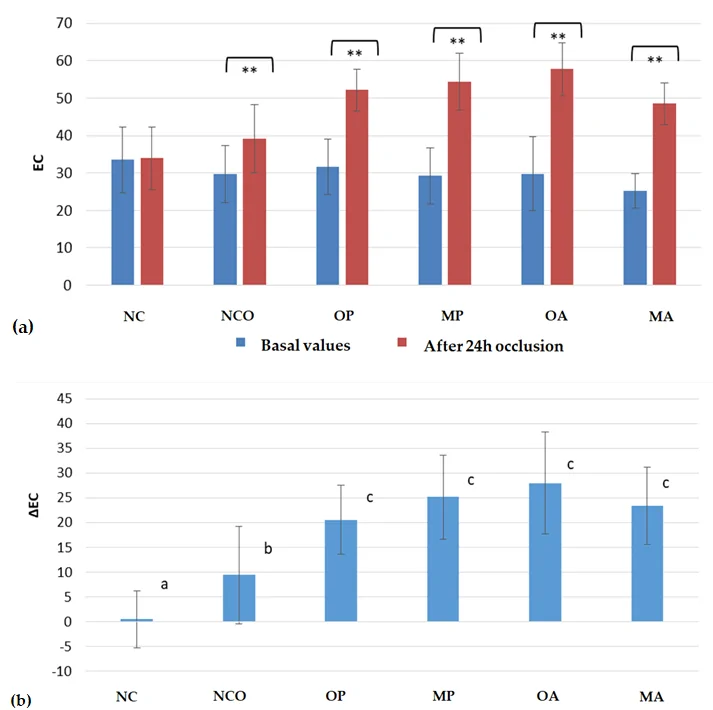
(**a**) Absolute values of EC recorded during an in vivo study evaluating the irritation potential of emulgels (OP, MP, OA, and MA) and untreated controls (NC and NCO). Results are presented as mean ± standard deviation, with statistically significant differences marked by symbol ** (*p* < 0.01). (**b**) Absolute values of ΔEC (changes relative to baseline values). Statistically significant differences between group means (*p* < 0.05) are indicated by different letters next to bars (a–c).

**Figure 4:**
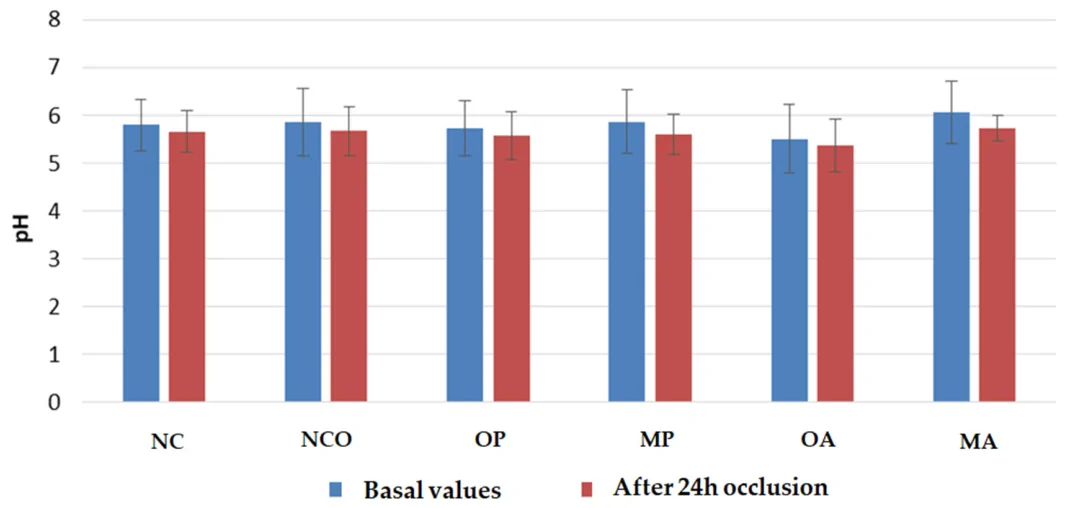
Absolute values of pH recorded during an in vivo study evaluating the irritation potential of emulgels (OP, MP, OA, and MA) and untreated controls (NC and NCO). Results are presented as mean ± standard deviation.

**Figure 5:**
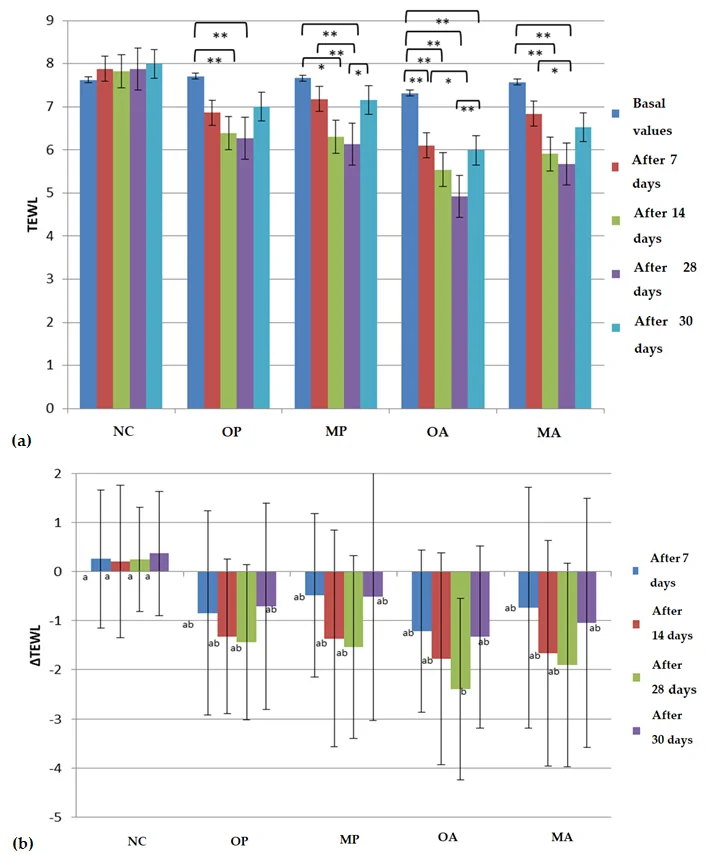
Assessment of TEWL after long-term in vivo application of emulgels (OP, MP, OA, and MA) versus an untreated control (NC). (**a**) Absolute values of TEWL, expressed as mean ± standard deviation, * (*p* < 0.05) and ** (*p* < 0.01). (**b**) Absolute changes in TEWL relative to baseline (ΔTEWL). Statistically significant differences between group means (*p* < 0.05) are indicated by different letters next to bars (a,b).

**Figure 6:**
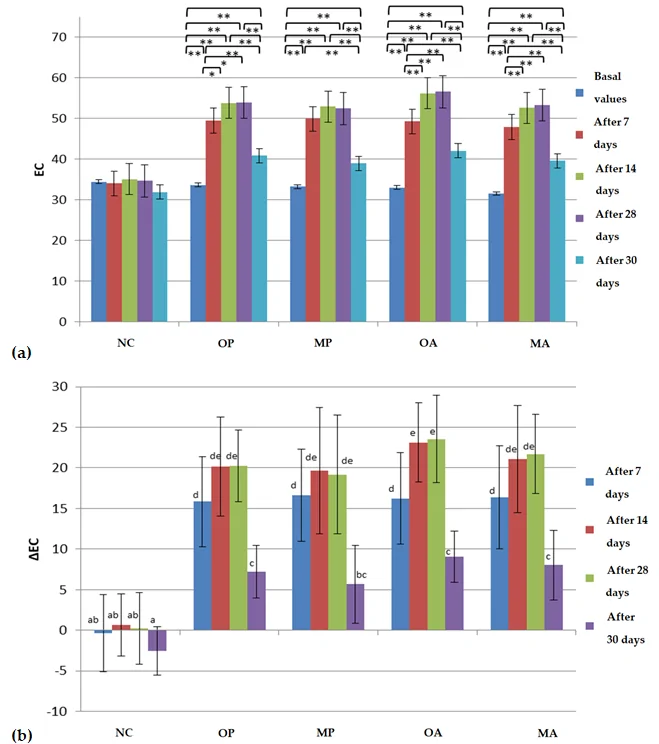
Assessment of EC after long-term in vivo application of emulgels (OP, MP, OA, and MA) versus an untreated control (NC). (**a**) Absolute values of EC, expressed as mean ± standard deviation, * (*p* < 0.05) and ** (*p* < 0.01). (**b**) Absolute changes in EC relative to baseline (ΔEC). Statistically significant differences between group means (*p* < 0.05) are indicated by different letters next to bars (a–e).

**Figure 7:**
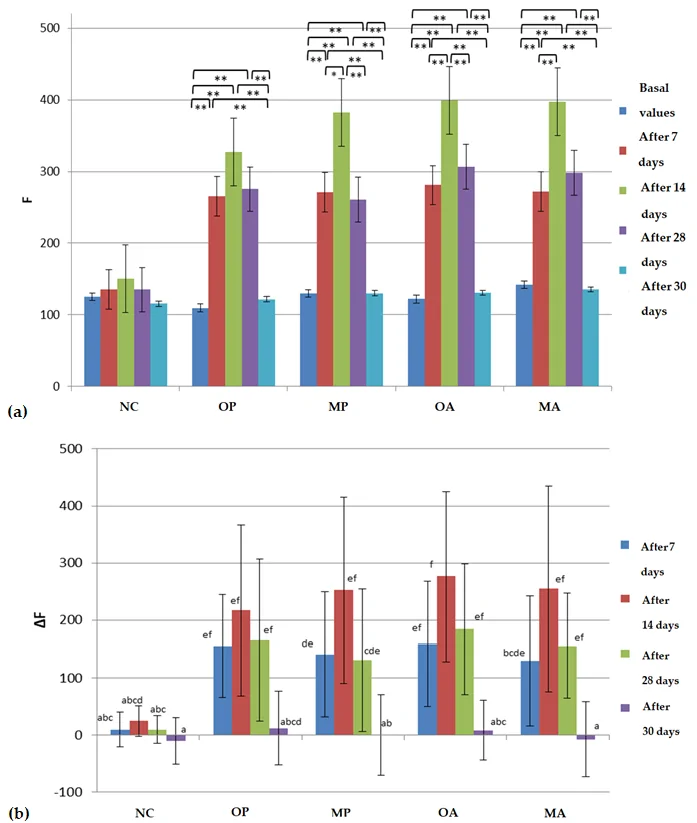
Assessment of F after long-term in vivo application of emulgels (OP, MP, OA, and MA) versus an untreated control (NC). (**a**) Absolute values of F, expressed as mean ± standard deviation, * (*p* < 0.05) and ** (*p* < 0.01). (**b**) Absolute changes in F relative to baseline (ΔF). Statistically significant differences between group means (*p* < 0.05) are indicated by different letters next to bars (a–f).

**Figure 8:**
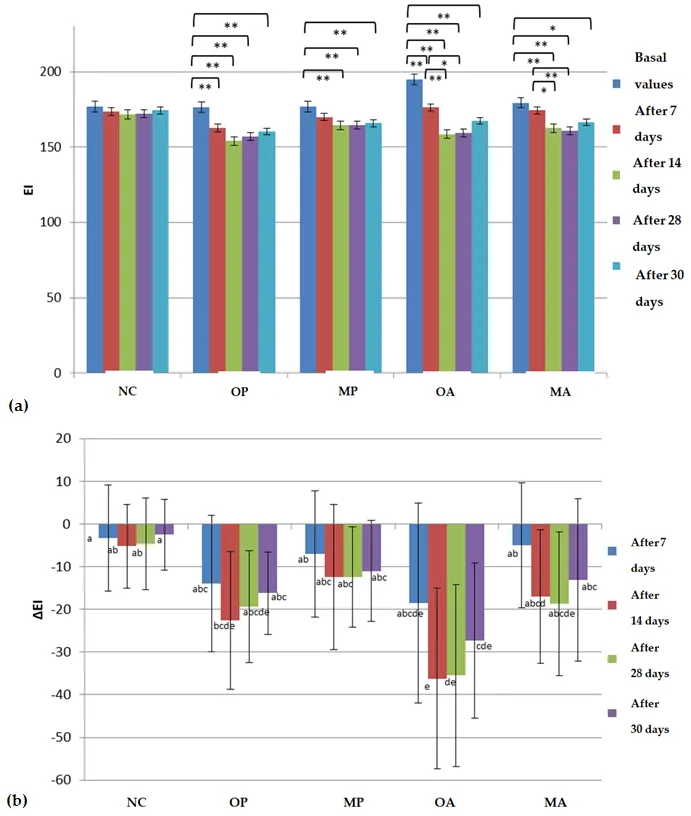
Assessment of EI after long-term in vivo application of emulgels (OP, MP, OA, and MA) versus an untreated control (NC). (**a**) Absolute values of EI, expressed as mean ± standard deviation, * (*p* < 0.05) and ** (*p* < 0.01). (**b**) Absolute changes in EI relative to baseline (ΔEI). Statistically significant differences between group means (*p* < 0.05) are indicated by different letters next to bars (a–e).

**Figure 9:**
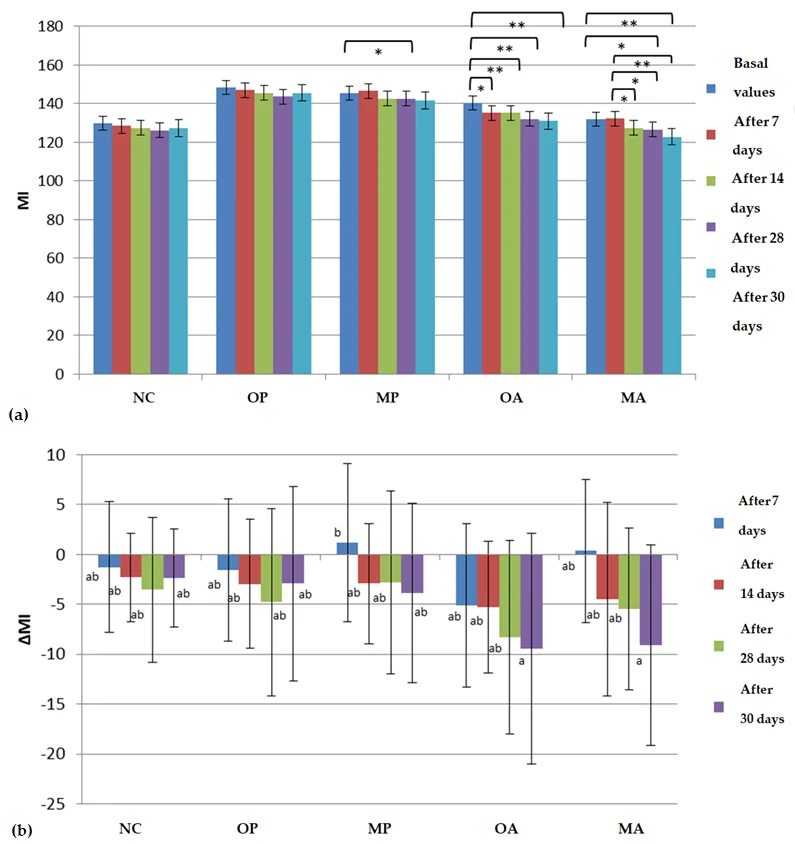
Assessment of MI after long-term in vivo application of emulgels (OP, MP, OA, and MA) versus an untreated control (NC). (**a**) Absolute values of MI, expressed as mean ± standard deviation, * (*p* < 0.05) and ** (*p* < 0.01). (**b**) Absolute changes in MI relative to baseline (ΔMI). Statistically significant differences between group means (*p* < 0.05) are indicated by different letters next to bars (a,b).

**Figure 10:**
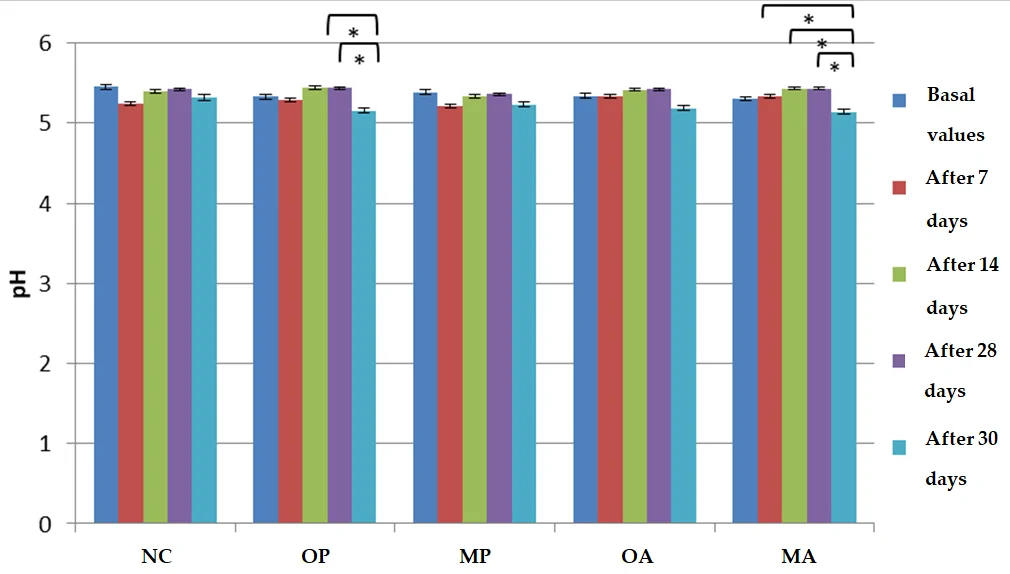
Assessment of skin barrier parameters after long-term in vivo application of emulgels (OP, MP, OA, and MA) versus an untreated control (NC). Absolute values of pH, expressed as mean ± standard deviation, * (*p* < 0.05).

**Figure 11:**
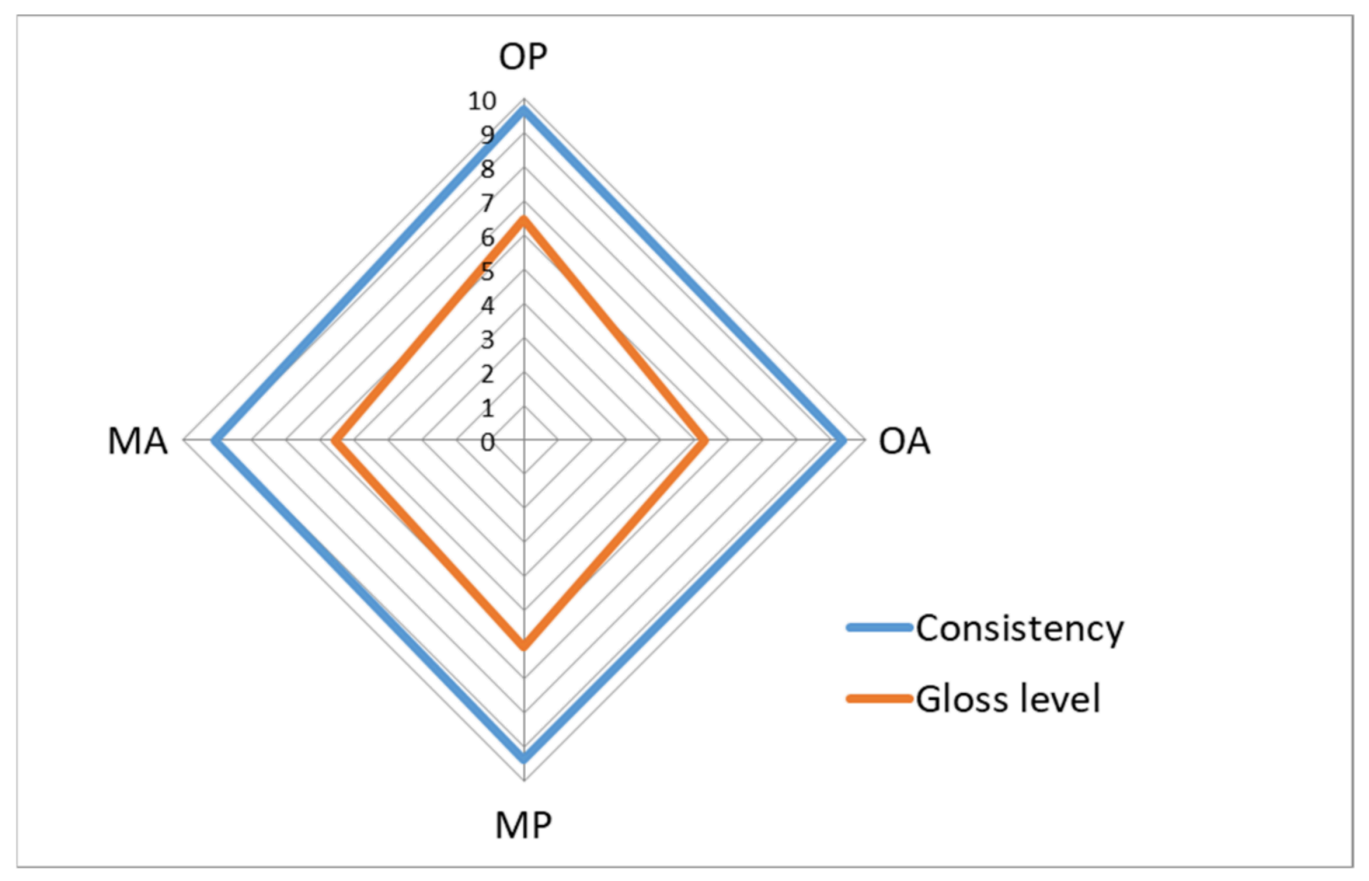
Results of the sensory evaluation of emulgels (OP, MP, OA, and MA) before application.

**Figure 12:**
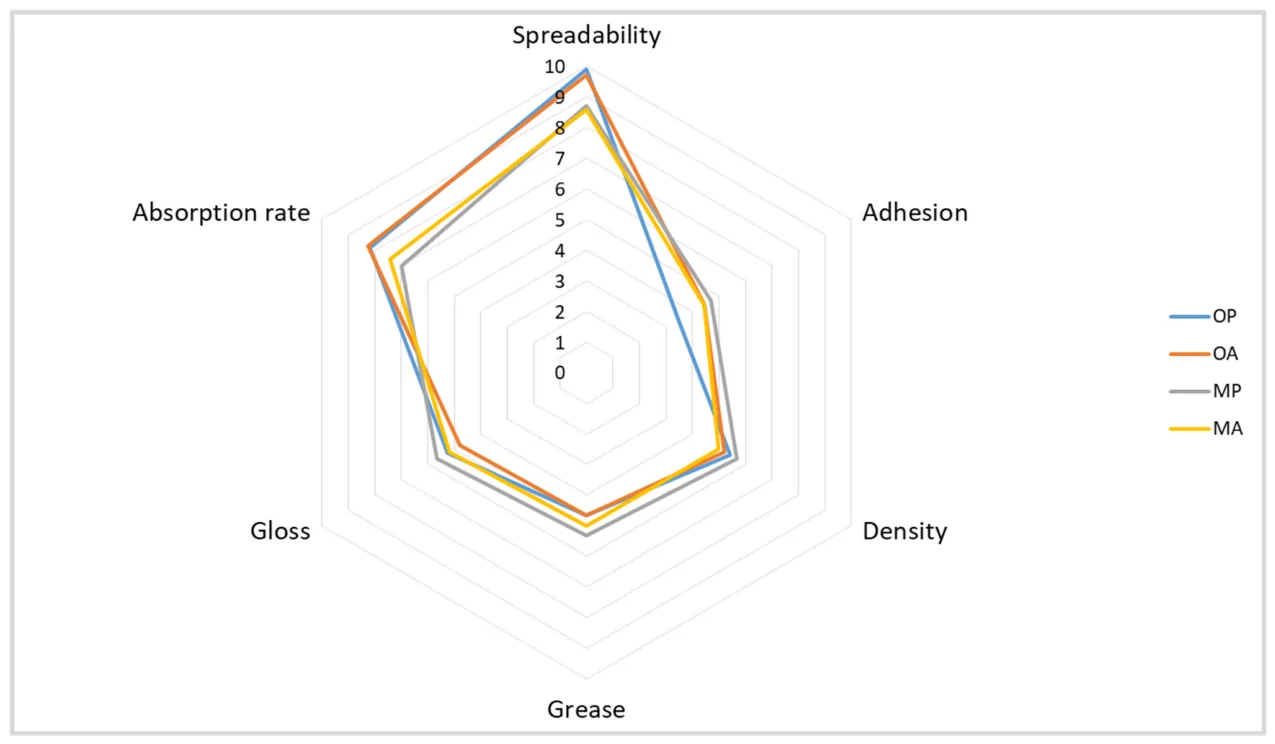
Results of the sensory evaluation of emulgels (OP, MP, OA, and MA) during the application.

**Figure 13:**
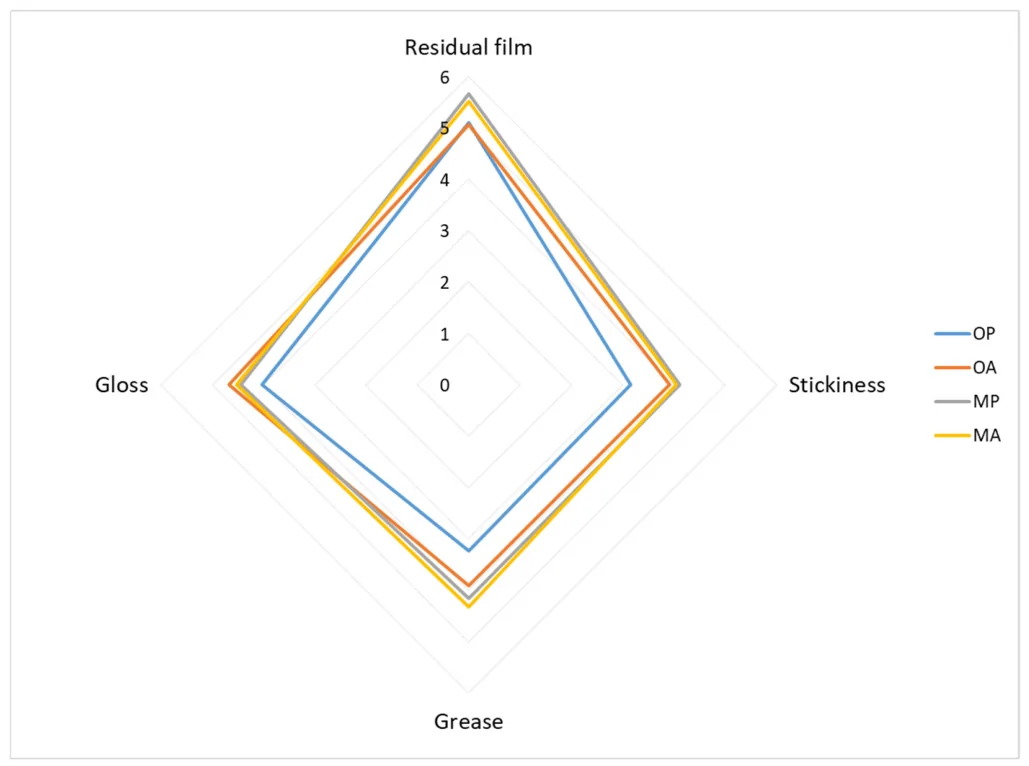
Results of the sensory evaluation of emulgels (OP, MP, OA, and MA) 10 min after the application.

**Figure 14:**
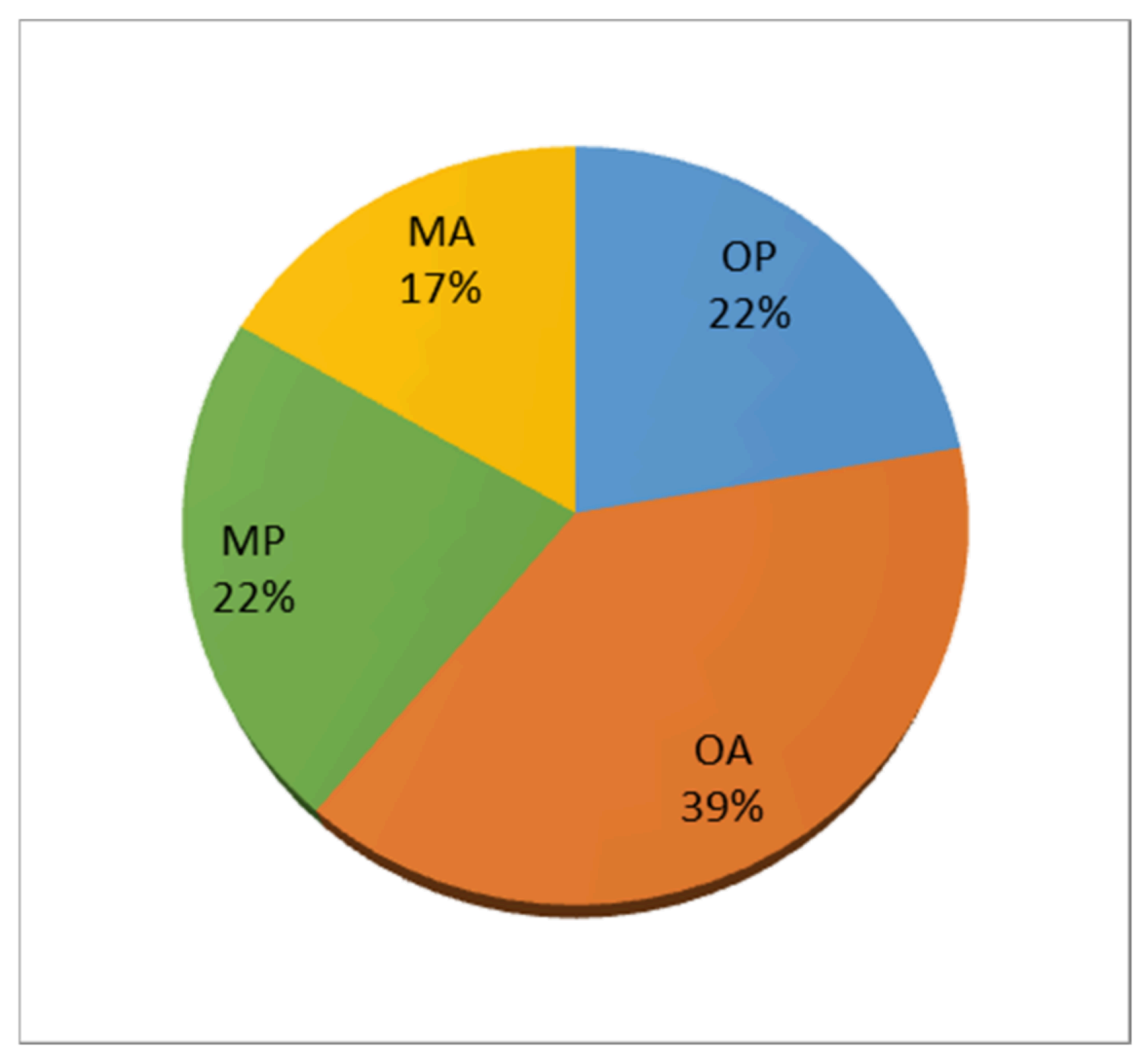
Answers to the question on participants’ overall product preference.

**Figure 15:**
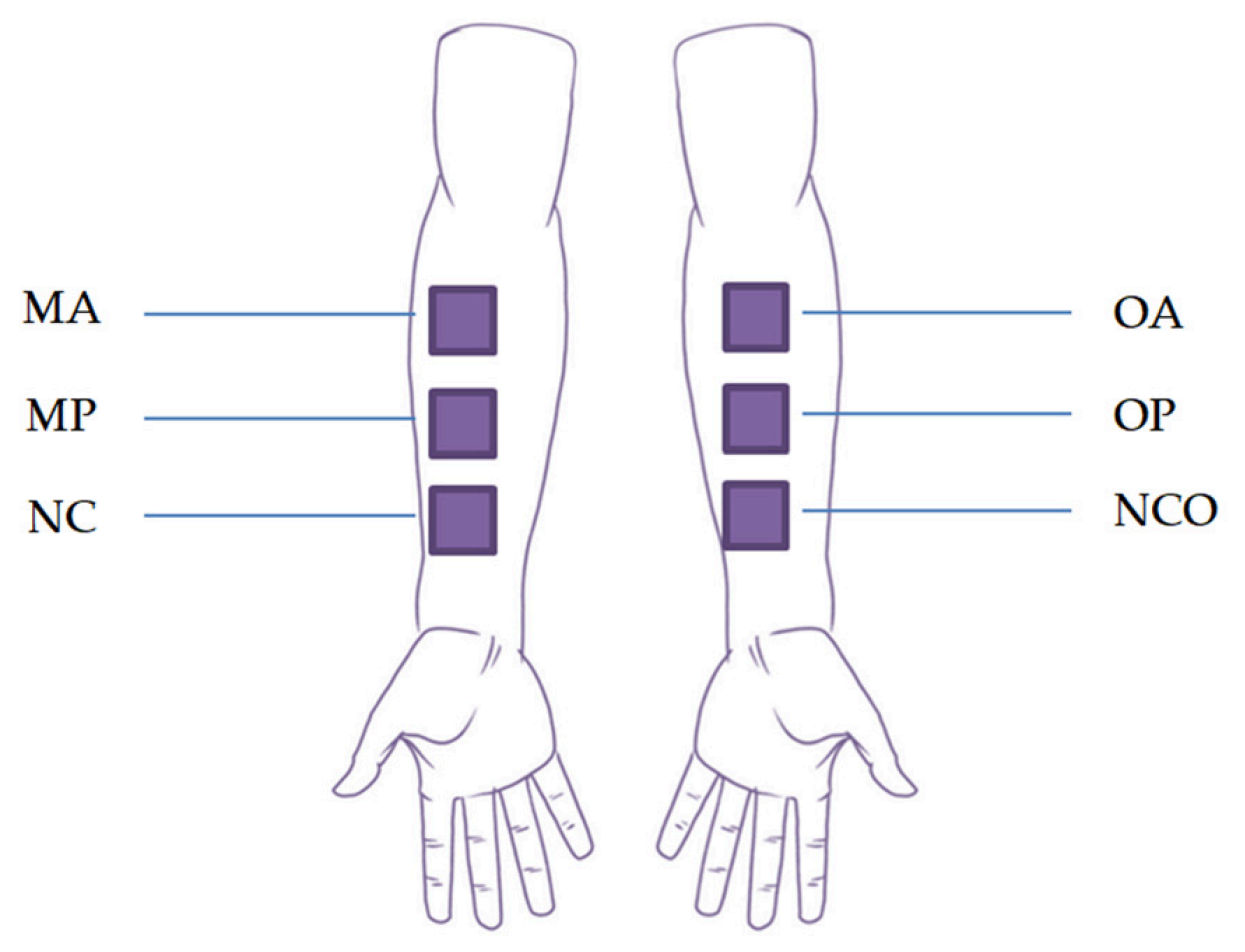
Diagram of the volar forearm illustrating the marked test application sites (MA, MP, OA and OP) and control sites (NC and NCO).

**Table 1:** Textural characteristics of emulgels MP, MA, OP, and OA.

	MP		MA		OP		OA	
	Basal	After 30 Days	Basal	After 30 Days	Basal	After 30 Days	Basal	After 30 Days
Hardness (mN)	367.50 ± 7.78	475.50 ± 34.65	985.50 ± 62.93	1247.50 ± 24.75	235.50 ± 13.44	429.00 ± 2.83	466.00 ± 7.07	875.50 ± 34.65
Adhesiveness (mJ)	0.70 ± 0.14	0.80 ± 0.28	1.40 ± 0.28	1.50 ± 0.28	0.60 ± 0.20	0.70 ± 0.28	1.60 ± 0.20	1.70 ± 0.28
Springiness (mm)	4.80 ± 0.03	4.24 ± 0.25	4.75 ± 0.29	4.51 ± 0.42	4.81 ± 0.20	4.67 ± 0.10	4.85 ± 0.49	4.90 ± 0.14
Resilience (a.u.)	0.03 ± 0.02	0.03 ± 0.01	0.02 ± 0.01	0.02 ± 0.01	0.02 ± 0.01	0.03 ± 0.01	0.03 ± 0.02	0.03 ± 0.02
Cohesiveness (a.u.)	0.65 ± 0.04	0.85 ± 0.10	0.75 ± 0.08	1.02 ± 0.06	0.67 ± 0.03	0.81 ± 0.10	0.78 ± 0.06	1.02 ± 0.07

**Table 2:** Information about plant materials used in the study.

	Plant Material	Latin Name of the Plant	Origin	Details of Identification and Voucher Number	
BL	Dried bilberry leaves	*Myrtilli folium*, *Vaccinium myrtillus* L., Ericaceae	Institute for Medicinal Plant Research “Dr. Josif Pančić” from Belgrade	VML_0921	Herbarium of Faculty of Pharmacy, University of Belgrade (Belgrade, Serbia).
TL	Dried green tea leaves	*Camelliae sinensis non fermentatum folium*, *Camellia sinensis* (L.) Kuntze, Theaceae		CSNonF_1121	

**Table 3:** Composition of emulgels used in the in vivo study.

INCI Name	Commercial Name	Supplier	MP	MA	OP	OA	Function
Cetearyl alcohol and cetearyl glucoside	Montanov™ 68MB	Seppic (La Garenne-Colombes, France)	6	6	-	-	Emulsifier
Sorbitan olivate and cetearyl olivate	Olivem^®^ 1000	Hallstar (Chicago, IL, USA)	-	-	5	5	Emulsifier
Cetearyl alcohol and cetyl palmitate and sorbitan palmitate and sorbitan oleate	Olivem^®^ VS Feel		-	-	1	1	Co-emulsifier
Caprylic/capric triglycerides	Myritol^®^ 318	BASF (Ludwigshafen, Germany)	20	20	20	20	Emollient
Glycerol	Glycerol	Comcen (Belgrade, Serbia)	8	8	8	8	Humectant
Potassium sorbate	Potassium sorbate	Comcen (Belgrade, Serbia)	1	1	1	1	Preservative
NaDES extracts (BL-MG + TL-TS)	not applicable		-	12	-	12	Active ingredient
Sodium hydroxide, 10%	Sodium hydroxide	Comcen (Belgrade, Serbia)	q.s. to adjust pH to 4.5				pH regulator
Hydroxyethyl cellulose	Hydroxyethyl cellulose	Chem Point (Krakow, Poland)	0.5	0.5	0.5	0.5	Gelling agent
Purified water	/	Faculty of Medicine (University of Niš, Niš, Serbia)	q.s. ad 100 *				Solvent

* Quantum satis ad, meaning “a sufficient quantity up to”.

**Table 4:** Parameters used for calculation of participants for the in vivo study.

Parameters	Value	Parameters	Value
effect size	f = 0.4	number of measurements	2 or 5
type I error probability	α = 0.05	correlation between repeated measures	0.5
study power	1-β = 0.8	non-sphericity correction for five measurements	ε = 0.75
number of groups	1	non-sphericity correction for two measurements	ε = 1

**Table 5:** The description of probes attached to the Multi Probe Adapter System MPA^®^ 9 (Courage + Khazaka Electronic GmbH, Ehrenfeld, Germany).

Probe	Measured Parameter	Units	Explanation
Corneometer^®^ CM 825	electrical capacitance of the skin (EC)	arbitrary units	skin hydration
Tewameter^®^ TM 300	transepidermal water loss (TEWL)	g/h/m^2^	skin barrier function
Mexameter^®^ MX 18	melanin index (MI) and erythema index (EI)	arbitrary units	skin pigmentation and redness
Skin-pH-Meter^®^ PH 905	pH	arbitrary units	skin surface pH
FrictionMeter FR 700	friction coefficient (F)	arbitrary units	skin friction and smoothness

**Table 6:** The description of attributes used in different phases of the sensory study.

Phase	Attribute	Description
Before application	Consistency (Viscosity)	Ease of dispensing the product from the packaging.
	Shine	The level of shine of the sample in the packaging.
During application	Spreadability	Degree of spreadability/slipperiness of the sample during application using two circular motions on the back of the hand.
	Stickiness	Degree of stickiness during application—the force required to separate fingers from the skin.
	Density	Perceived density of the sample during application.
	Oily Feel	Degree of oiliness felt during application.
	Shine	The extent to which the sample appears shiny when applied to the skin.
	Absorption Rate	The speed at which the sample is absorbed into the skin.
After application	Residual Film	Amount of residual film left on the skin 10 min after application.
	Stickiness	Degree of stickiness felt on the skin 10 min after application.
	Oily Feel	Degree of oiliness felt on the skin 10 min after application.
	Shine	The level of shine observed on the skin 10 min after application.

**Table 7:** Overview of attributes used in the sensory study and their quantitative features.

Attribute	1	2	3	4	5
Consistency	Liquid	Semi-solid	/	/	/
Shine	Matte	Pearly shine	Slightly shiny	Shiny	Very shiny
Spreadability	Very difficult to spread	Difficult to spread	Easy to spread	/	/
Stickiness	Not sticky	Slightly sticky	Sticky	Very sticky	/
Density	Thin	Slightly dense	Dense	Very dense	/
Oily Feel	Not oily	Slightly oily	Oily	Very oily	/
Absorption Rate	Slow	Moderate	Fast	/	/
Residual Film	No film	Moderate film	Pronounced film	/	/

## Data Availability

The original contributions presented in this study are included in the article. Further inquiries can be directed to the corresponding author.

## Supplementary Materials

The following supporting information can be downloaded at: https://www.mdpi.com/article/10.3390/gels12100870/s1. Table S1: Statistical parameters of three-way repeated-measures ANOVA following a 2x2x5 factorial design of factors Emulsifier—sorbitan/cetearyl olivate or cetearyl alcohol/glucoside x Extracts, indicating presence of extract—placebo or active emulgel x Time – basal, day 7, day 14, day 28, day 30..

## Abbreviations

The following abbreviations are used in this manuscript:

TEWL	Transepidermal water loss
EI	Erythema index
EC	Skin capacitance
MI	Melanin index
F	Friction coefficient
NaDES	Natural deep eutectic solvent
HEC	Hydroxyethyl cellulose
NC	Untreated control site
NCO	Untreated control site under occlusion
TL	Dried green tea leaves
BL	Dried bilberry leaves
MG	NaDES composed of malic acid-glycerol (1:2 mol/mol)
TS	NaDES composed of tartaric acid-sorbitol (1:2 mol/mol)
