# Supplementary material for: HEC-Based Emulgels as Vehicles for NaDES Extracts: Safety, Efficacy, and Sensory Evaluation
Source: Gels. 2026 Sep 25;12(10):870. doi: 10.3390/gels12100870 (PMC13704478; doi:10.3390/gels12100870)
Supplement: Supplementary file 1 [file gels-12-00870-s001.zip › gels-4536219-supplementary.pdf]

Supplementary Material

HEC-Based Emulgels as Vehicles for NaDES Extracts: Safety, Efficacy, and Sensory Evaluation

Milica Martinović [1,*], Vanja M. Tadić [2,*], Ana Žugić [2], Slavica Blagojević [3], Stevan Blagojević [4] and Ivana Nešić [1]

[1] Department of Pharmacy, Faculty of Medicine, University of Niš, Boulevard Dr. Zorana Djindjića 81, 18108 Niš, Serbia; ivana.nesic@medfak.ni.ac.rs

[2] Department for Pharmaceutical Research and Development, Institute for Medicinal Plant Research "Dr. Josif Pančić", Tadeuša Koscuška 1, 11000 Belgrade, Serbia; azugic@mocbilja.rs

[3] Faculty of Pharmacy, University of Belgrade, Vojvode Stepe 450, 11221 Belgrade, Serbia; slavica.blagojevic@pharmacy.bg.ac.rs

[4] The Institute of General and Physical Chemistry, Studentski trg 12/V, 11158 Beograd, Serbia; sblagojevic@iofh.bg.ac.rs

* Correspondence: milica.martinovic@medfak.ni.ac.rs (M.M.); vtadic@mocbilja.rs (V.M.T.)

Table S1. Statistical parameters of three-way repeated-measures ANOVA following a 2x2x5 factorial design of factors Emulsifier - sorbitan/cetearyl olivate or cetearyl alcohol/glucoside x Extracts, indicating presence of extract – placebo or active emulgel x Time – basal, day 7, day 14, day 28, day 30.

Parameter	Effect	Degrees of Freedom	F	p	η_p^2	Statistical Significance
TEWL (Transepidermal Water Loss)	Main Effects					
	Emulsifier	1, 16	3.084	0.098	0.162	no
	Extract	1, 16	1.713	0.209	0.097	no
	Time	4, 64	8.392	< 0.001	0.344	p<0.001
	Interaction Effects					
	Emulsifier × Extract	1, 16	12.044	0.003	0.429	p<0.01
	Extract × Time	4, 64	0.314	0.867	0.019	no
	Emulsifier × Time	4, 64	0.143	0.965	0.009	no
	Emulsifier × Extract × Time	4, 64	1.016	0.406	0.06	no
EC (Electrical Capacitance)	Main Effects					
	Emulsifier	1, 16	2.168	0.16	0.119	no
	Extract	1, 16	4.903	0.042	0.235	p<0.05
	Time	4, 64	128.698	< 0.001	0.889	p<0.001
	Interaction Effects					
	Emulsifier ×	1, 16	0.205	0.657	0.013	no

	Extract					
	Extract × Time	4, 64	1.054	0.386	0.062	no
	Emulsifier × Time	4, 64	0.29	0.883	0.018	no
	Emulsifier × Extract × Time	4, 64	3.319	0.016	0.172	p<0.05
F (Friction Coefficient)	Main Effects					
	Emulsifier	1, 16	0.61	0.446	0.037	no
	Extract	1, 16	0.895	0.358	0.053	no
	Time	4, 64	34.267	< 0.001	0.682	p<0.001
	Interaction Effects					
	Emulsifier × Extract	1, 16	7.208	0.016	0.311	p<0.05
	Extract × Time	4, 64	1.111	0.343	0.065	no
	Emulsifier × Time	4, 64	0.943	0.424	0.056	no
	Emulsifier × Extract × Time	4, 64	1.553	0.198	0.088	no
EI (Erythema Index)	Main Effects					
	Emulsifier	1, 16	1.854	0.192	0.104	no
	Extract	1, 16	0.145	0.709	0.009	no
	Time	4, 64	17.71	< 0.001	0.525	p<0.001
	Interaction Effects					
	Emulsifier × Extract	1, 16	1.301	0.271	0.075	no
	Extract × Time	4, 64	3.293	0.016	0.171	p<0.05
	Emulsifier × Time	4, 64	0.673	0.613	0.04	no
	Emulsifier × Extract × Time	4, 64	3.708	0.009	0.188	p<0.01
MI (Melanin Index)	Main Effects					
	Emulsifier	1, 16	3.73	0.071	0.189	no
	Extract	1, 16	3.375	0.085	0.174	no
	Time	4, 64	7.918	< 0.001	0.331	p<0.001
	Interaction Effects					
	Emulsifier × Extract	1, 16	49.737	< 0.001	0.757	p<0.001
	Extract ×	4, 64	1.732	0.154	0.098	no

	Time					
	Emulsifier × Time	4, 64	0.137	0.915	0.008	no
	Emulsifier × Extract × Time	4, 64	2.084	0.116	0.115	no
pH	Main Effects					
	Emulsifier	1, 16	0.105	0.751	0.006	no
	Extract	1, 16	0.603	0.449	0.036	no
	Time	4, 64	2.148	0.085	0.118	no
	Interaction Effects					
	Emulsifier × Extract	1, 16	0.372	0.55	0.023	no
	Emulsifier × Time	4, 64	3.633	0.01	0.185	$p<0.05$
	Extract × Time	4, 64	0.328	0.858	0.02	no
	Emulsifier × Extract × Time	4, 64	1.51	0.21	0.086	no
